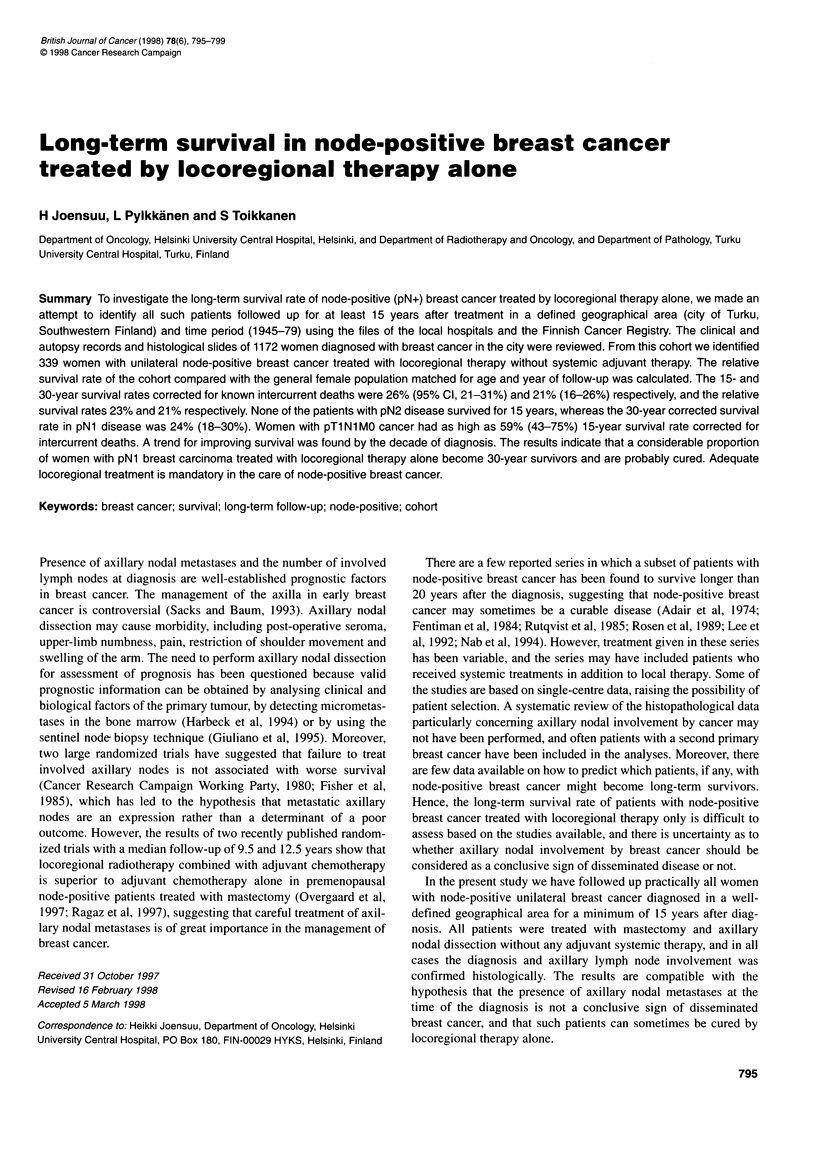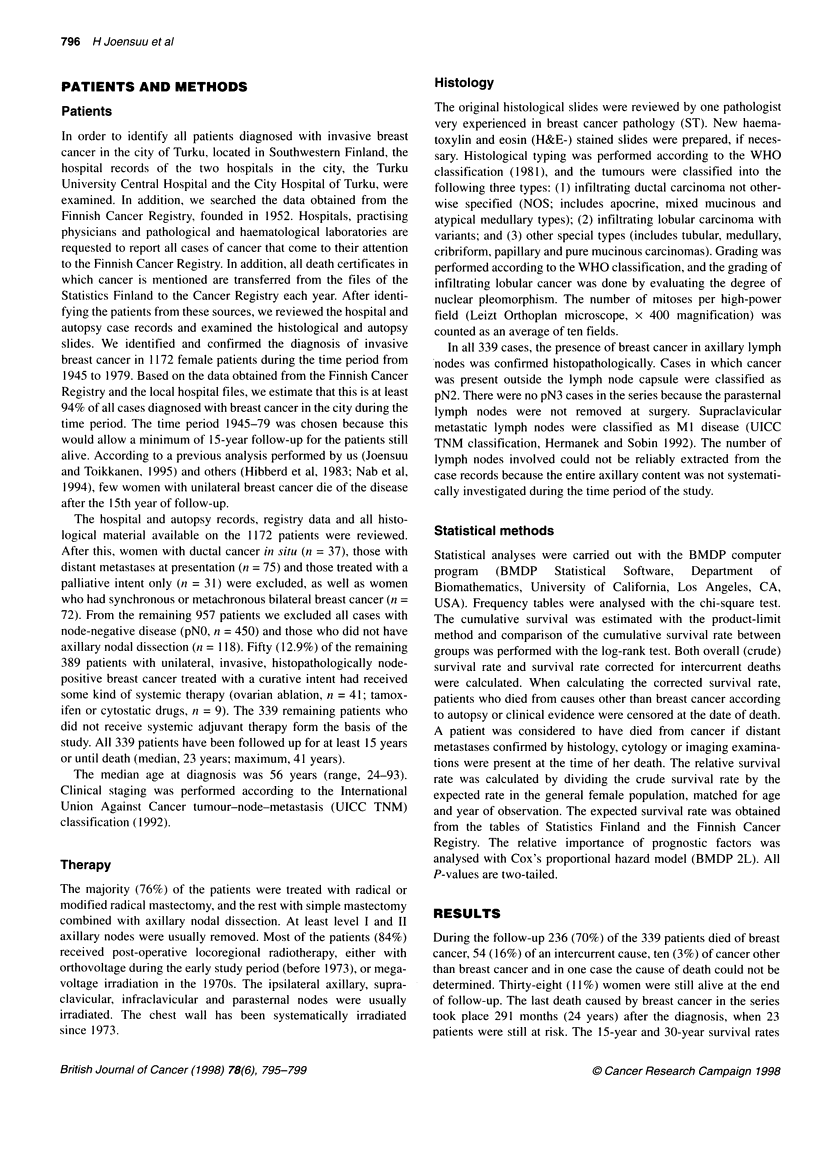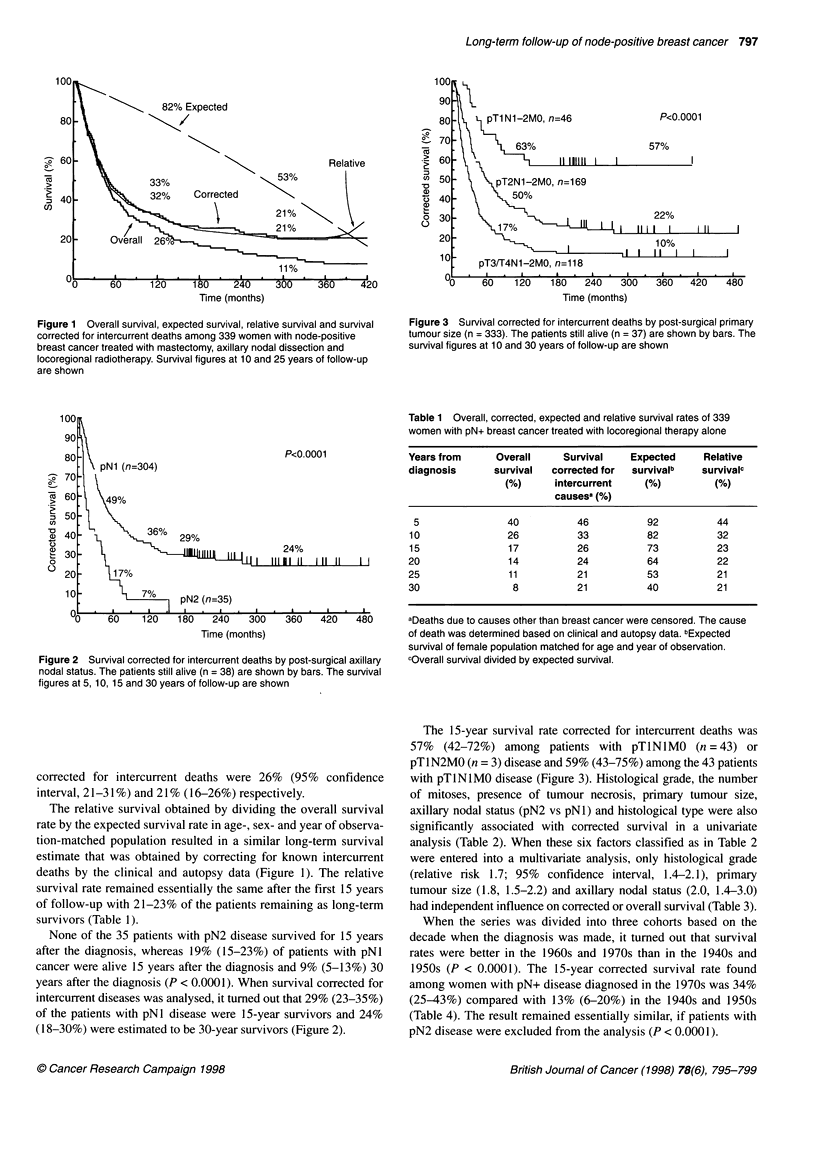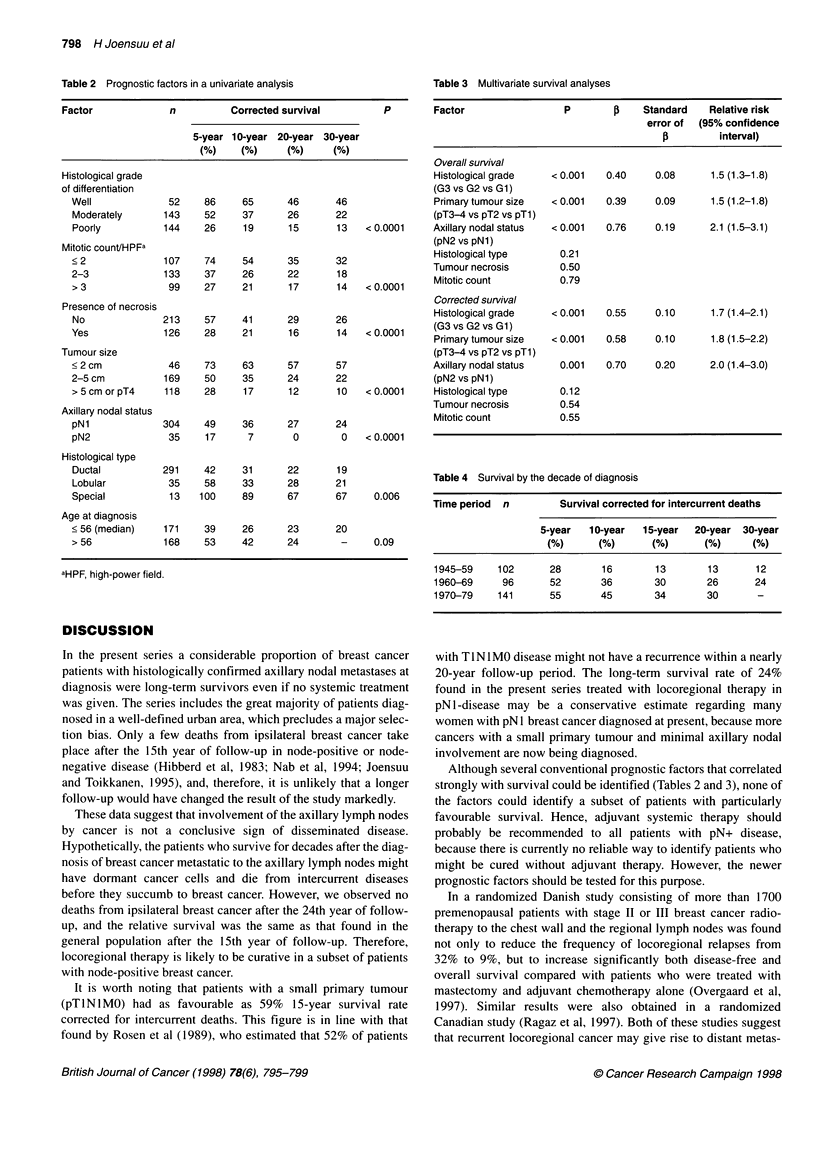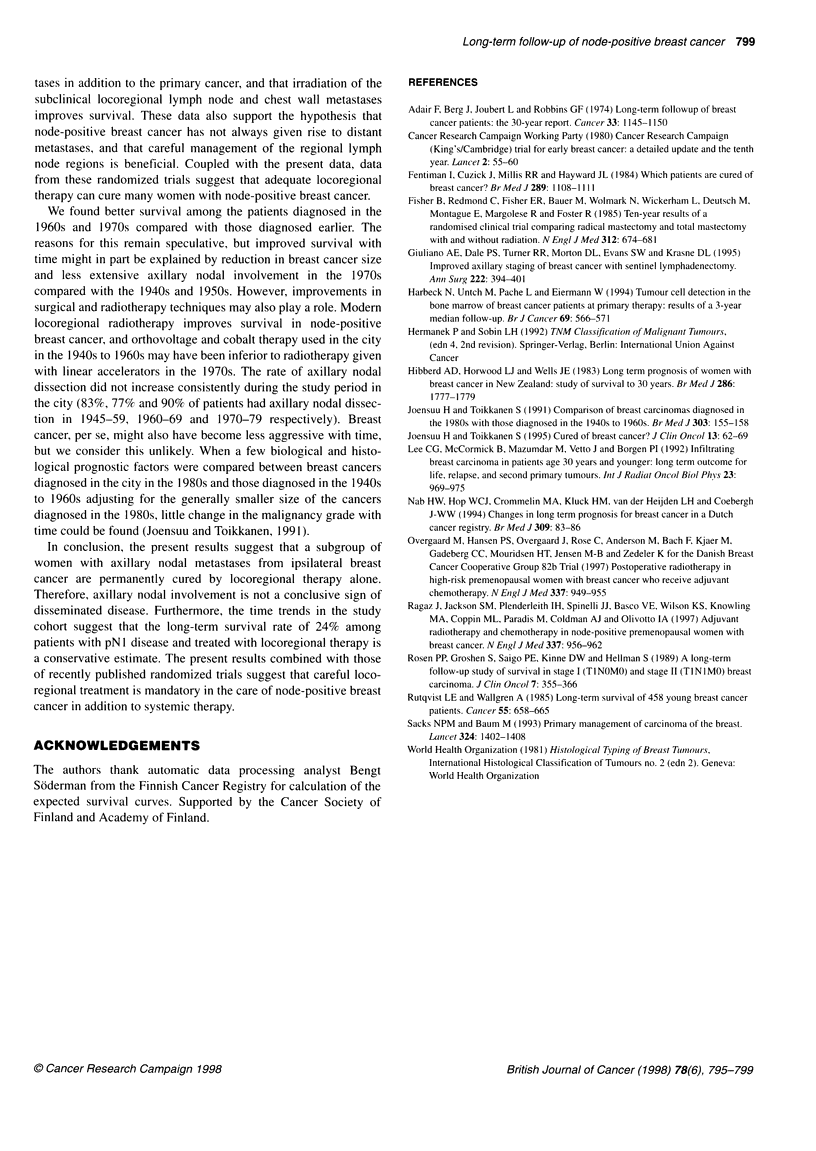# Long-term survival in node-positive breast cancer treated by locoregional therapy alone.

**DOI:** 10.1038/bjc.1998.581

**Published:** 1998-09

**Authors:** H. Joensuu, L. Pylkkänen, S. Toikkanen

**Affiliations:** Department of Oncology, Helsinki University Central Hospital, Finland.

## Abstract

To investigate the long-term survival rate of node-positive (pN+) breast cancer treated by locoregional therapy alone, we made an attempt to identify all such patients followed up for at least 15 years after treatment in a defined geographical area (city of Turku, Southwestern Finland) and time period (1945-79) using the files of the local hospitals and the Finnish Cancer Registry. The clinical and autopsy records and histological slides of 1172 women diagnosed with breast cancer in the city were reviewed. From this cohort we identified 339 women with unilateral node-positive breast cancer treated with locoregional therapy without systemic adjuvant therapy. The relative survival rate of the cohort compared with the general female population matched for age and year of follow-up was calculated. The 15- and 30-year survival rates corrected for known intercurrent deaths were 26% (95% CI, 21-31%) and 21% (16-26%) respectively, and the relative survival rates 23% and 21% respectively. None of the patients with pN2 disease survived for 15 years, whereas the 30-year corrected survival rate in pN1 disease was 24% (18-30%). Women with pT1N1M0 cancer had as high as 59% (43-75%) 15-year survival rate corrected for intercurrent deaths. A trend for improving survival was found by the decade of diagnosis. The results indicate that a considerable proportion of women with pN1 breast carcinoma treated with locoregional therapy alone become 30-year survivors and are probably cured. Adequate locoregional treatment is mandatory in the care of node-positive breast cancer.


British Journal of Cancer (1998) 78(6), 795–799
© 1998 Cancer Research Campaign

### Long-term survival in node-positive breast cancer treated by locoregional therapy alone

**H Joensuu, L Pylkkänen and S Toikkanen**

Department of Oncology, Helsinki University Central Hospital, Helsinki, and Department of Radiotherapy and Oncology, and Department of Pathology, Turku University Central Hospital, Turku, Finland

**Summary** To investigate the long-term survival rate of node-positive (pN+) breast cancer treated by locoregional therapy alone, we made an attempt to identify all such patients followed up for at least 15 years after treatment in a defined geographical area (city of Turku, Southwestern Finland) and time period (1945–79) using the files of the local hospitals and the Finnish Cancer Registry. The clinical and autopsy records and histological slides of 1172 women diagnosed with breast cancer in the city were reviewed. From this cohort we identified 339 women with unilateral node-positive breast cancer treated with locoregional therapy without systemic adjuvant therapy. The relative survival rate of the cohort compared with the general female population matched for age and year of follow-up was calculated. The 15- and 30-year survival rates corrected for known intercurrent deaths were 26% (95% CI, 21–31%) and 21% (16–26%) respectively, and the relative survival rates 23% and 21% respectively. None of the patients with pN2 disease survived for 15 years, whereas the 30-year corrected survival rate in pN1 disease was 24% (18–30%). Women with pT1N1M0 cancer had as high as 59% (43–75%) 15-year survival rate corrected for intercurrent deaths. A trend for improving survival was found by the decade of diagnosis. The results indicate that a considerable proportion of women with pN1 breast carcinoma treated with locoregional therapy alone become 30-year survivors and are probably cured. Adequate locoregional treatment is mandatory in the care of node-positive breast cancer.

**Keywords:** breast cancer; survival; long-term follow-up; node-positive; cohort

Presence of axillary nodal metastases and the number of involved lymph nodes at diagnosis are well-established prognostic factors in breast cancer. The management of the axilla in early breast cancer is controversial (Sacks and Baum, 1993). Axillary nodal dissection may cause morbidity, including post-operative seroma, upper-limb numbness, pain, restriction of shoulder movement and swelling of the arm. The need to perform axillary nodal dissection for assessment of prognosis has been questioned because valid prognostic information can be obtained by analysing clinical and biological factors of the primary tumour, by detecting micrometastases in the bone marrow (Harbeck et al, 1994) or by using the sentinel node biopsy technique (Giuliano et al, 1995). Moreover, two large randomized trials have suggested that failure to treat involved axillary nodes is not associated with worse survival (Cancer Research Campaign Working Party, 1980; Fisher et al, 1985), which has led to the hypothesis that metastatic axillary nodes are an expression rather than a determinant of a poor outcome. However, the results of two recently published randomized trials with a median follow-up of 9.5 and 12.5 years show that locoregional radiotherapy combined with adjuvant chemotherapy is superior to adjuvant chemotherapy alone in premenopausal node-positive patients treated with mastectomy (Overgaard et al, 1997; Ragaz et al, 1997), suggesting that careful treatment of axillary nodal metastases is of great importance in the management of breast cancer.

*Received 31 October 1997*
*Revised 16 February 1998*
*Accepted 5 March 1998*

*Correspondence to:* Heikki Joensuu, Department of Oncology, Helsinki University Central Hospital, PO Box 180, FIN-00029 HYKS, Helsinki, Finland

There are a few reported series in which a subset of patients with node-positive breast cancer has been found to survive longer than 20 years after the diagnosis, suggesting that node-positive breast cancer may sometimes be a curable disease (Adair et al, 1974; Fentiman et al, 1984; Rutqvist et al, 1985; Rosen et al, 1989; Lee et al, 1992; Nab et al, 1994). However, treatment given in these series has been variable, and the series may have included patients who received systemic treatments in addition to local therapy. Some of the studies are based on single-centre data, raising the possibility of patient selection. A systematic review of the histopathological data particularly concerning axillary nodal involvement by cancer may not have been performed, and often patients with a second primary breast cancer have been included in the analyses. Moreover, there are few data available on how to predict which patients, if any, with node-positive breast cancer might become long-term survivors. Hence, the long-term survival rate of patients with node-positive breast cancer treated with locoregional therapy only is difficult to assess based on the studies available, and there is uncertainty as to whether axillary nodal involvement by breast cancer should be considered as a conclusive sign of disseminated disease or not.

In the present study we have followed up practically all women with node-positive unilateral breast cancer diagnosed in a well-defined geographical area for a minimum of 15 years after diagnosis. All patients were treated with mastectomy and axillary nodal dissection without any adjuvant systemic therapy, and in all cases the diagnosis and axillary lymph node involvement was confirmed histologically. The results are compatible with the hypothesis that the presence of axillary nodal metastases at the time of the diagnosis is not a conclusive sign of disseminated breast cancer, and that such patients can sometimes be cured by locoregional therapy alone.

### PATIENTS AND METHODS

### Patients

In order to identify all patients diagnosed with invasive breast cancer in the city of Turku, located in Southwestern Finland, the hospital records of the two hospitals in the city, the Turku University Central Hospital and the City Hospital of Turku, were examined. In addition, we searched the data obtained from the Finnish Cancer Registry, founded in 1952. Hospitals, practising physicians and pathological and haematological laboratories are requested to report all cases of cancer that come to their attention to the Finnish Cancer Registry. In addition, all death certificates in which cancer is mentioned are transferred from the files of the Statistics Finland to the Cancer Registry each year. After identifying the patients from these sources, we reviewed the hospital and autopsy case records and examined the histological and autopsy slides. We identified and confirmed the diagnosis of invasive breast cancer in 1172 female patients during the time period from 1945 to 1979. Based on the data obtained from the Finnish Cancer Registry and the local hospital files, we estimate that this is at least 94% of all cases diagnosed with breast cancer in the city during the time period. The time period 1945–79 was chosen because this would allow a minimum of 15-year follow-up for the patients still alive. According to a previous analysis performed by us (Joensuu and Toikkanen, 1995) and others (Hibberd et al, 1983; Nab et al, 1994), few women with unilateral breast cancer die of the disease after the 15th year of follow-up.

The hospital and autopsy records, registry data and all histological material available on the 1172 patients were reviewed. After this, women with ductal cancer *in situ* ($n$ = 37), those with distant metastases at presentation ($n$ = 75) and those treated with a palliative intent only ($n$ = 31) were excluded, as well as women who had synchronous or metachronous bilateral breast cancer ($n$ = 72). From the remaining 957 patients we excluded all cases with node-negative disease (pN0, $n$ = 450) and those who did not have axillary nodal dissection ($n$ = 118). Fifty (12.9%) of the remaining 389 patients with unilateral, invasive, histopathologically node-positive breast cancer treated with a curative intent had received some kind of systemic therapy (ovarian ablation, $n$ = 41; tamoxifen or cytostatic drugs, $n$ = 9). The 339 remaining patients who did not receive systemic adjuvant therapy form the basis of the study. All 339 patients have been followed up for at least 15 years or until death (median, 23 years; maximum, 41 years).

The median age at diagnosis was 56 years (range, 24–93). Clinical staging was performed according to the International Union Against Cancer tumour–node–metastasis (UICC TNM) classification (1992).

### Therapy

The majority (76%) of the patients were treated with radical or modified radical mastectomy, and the rest with simple mastectomy combined with axillary nodal dissection. At least level I and II axillary nodes were usually removed. Most of the patients (84%) received post-operative locoregional radiotherapy, either with orthovoltage during the early study period (before 1973), or megavoltage irradiation in the 1970s. The ipsilateral axillary, supraclavicular, infraclavicular and parasternal nodes were usually irradiated. The chest wall has been systematically irradiated since 1973.

### Histology

The original histological slides were reviewed by one pathologist very experienced in breast cancer pathology (ST). New haematoxylin and eosin (H&E-) stained slides were prepared, if necessary. Histological typing was performed according to the WHO classification (1981), and the tumours were classified into the following three types: (1) infiltrating ductal carcinoma not otherwise specified (NOS; includes apocrine, mixed mucinous and atypical medullary types); (2) infiltrating lobular carcinoma with variants; and (3) other special types (includes tubular, medullary, cribriform, papillary and pure mucinous carcinomas). Grading was performed according to the WHO classification, and the grading of infiltrating lobular cancer was done by evaluating the degree of nuclear pleomorphism. The number of mitoses per high-power field (Leizt Orthoplan microscope, × 400 magnification) was counted as an average of ten fields.

In all 339 cases, the presence of breast cancer in axillary lymph nodes was confirmed histopathologically. Cases in which cancer was present outside the lymph node capsule were classified as pN2. There were no pN3 cases in the series because the parasternal lymph nodes were not removed at surgery. Supraclavicular metastatic lymph nodes were classified as M1 disease (UICC TNM classification, Hermanek and Sobin 1992). The number of lymph nodes involved could not be reliably extracted from the case records because the entire axillary content was not systematically investigated during the time period of the study.

### Statistical methods

Statistical analyses were carried out with the BMDP computer program (BMDP Statistical Software, Department of Biomathematics, University of California, Los Angeles, CA, USA). Frequency tables were analysed with the chi-square test. The cumulative survival was estimated with the product-limit method and comparison of the cumulative survival rate between groups was performed with the log-rank test. Both overall (crude) survival rate and survival rate corrected for intercurrent deaths were calculated. When calculating the corrected survival rate, patients who died from causes other than breast cancer according to autopsy or clinical evidence were censored at the date of death. A patient was considered to have died from cancer if distant metastases confirmed by histology, cytology or imaging examinations were present at the time of her death. The relative survival rate was calculated by dividing the crude survival rate by the expected rate in the general female population, matched for age and year of observation. The expected survival rate was obtained from the tables of Statistics Finland and the Finnish Cancer Registry. The relative importance of prognostic factors was analysed with Cox's proportional hazard model (BMDP 2L). All $P$-values are two-tailed.

### RESULTS

During the follow-up 236 (70%) of the 339 patients died of breast cancer, 54 (16%) of an intercurrent cause, ten (3%) of cancer other than breast cancer and in one case the cause of death could not be determined. Thirty-eight (11%) women were still alive at the end of follow-up. The last death caused by breast cancer in the series took place 291 months (24 years) after the diagnosis, when 23 patients were still at risk. The 15-year and 30-year survival rates

© Cancer Research Campaign 1998

**Figure 1** Overall survival, expected survival, relative survival and survival corrected for intercurrent deaths among 339 women with node-positive breast cancer treated with mastectomy, axillary nodal dissection and locoregional radiotherapy. Survival figures at 10 and 25 years of follow-up are shown

**Figure 2** Survival corrected for intercurrent deaths by post-surgical axillary nodal status. The patients still alive (n = 38) are shown by bars. The survival figures at 5, 10, 15 and 30 years of follow-up are shown

**Figure 3** Survival corrected for intercurrent deaths by post-surgical primary tumour size (n = 333). The patients still alive (n = 37) are shown by bars. The survival figures at 10 and 30 years of follow-up are shown

**Table 1** Overall, corrected, expected and relative survival rates of 339 women with pN+ breast cancer treated with locoregional therapy alone

| Years from diagnosis | Overall survival (%) | Survival corrected for intercurrent causes[a] (%) | Expected survival[b] (%) | Relative survival[c] (%) |
|---|---|---|---|---|
| 5 | 40 | 46 | 92 | 44 |
| 10 | 26 | 33 | 82 | 32 |
| 15 | 17 | 26 | 73 | 23 |
| 20 | 14 | 24 | 64 | 22 |
| 25 | 11 | 21 | 53 | 21 |
| 30 | 8 | 21 | 40 | 21 |

[a]Deaths due to causes other than breast cancer were censored. The cause of death was determined based on clinical and autopsy data. [b]Expected survival of female population matched for age and year of observation. [c]Overall survival divided by expected survival.

corrected for intercurrent deaths were 26% (95% confidence interval, 21–31%) and 21% (16–26%) respectively.

The relative survival obtained by dividing the overall survival rate by the expected survival rate in age-, sex- and year of observation-matched population resulted in a similar long-term survival estimate that was obtained by correcting for known intercurrent deaths by the clinical and autopsy data (Figure 1). The relative survival rate remained essentially the same after the first 15 years of follow-up with 21–23% of the patients remaining as long-term survivors (Table 1).

None of the 35 patients with pN2 disease survived for 15 years after the diagnosis, whereas 19% (15–23%) of patients with pN1 cancer were alive 15 years after the diagnosis and 9% (5–13%) 30 years after the diagnosis ($P < 0.0001$). When survival corrected for intercurrent diseases was analysed, it turned out that 29% (23–35%) of the patients with pN1 disease were 15-year survivors and 24% (18–30%) were estimated to be 30-year survivors (Figure 2).

The 15-year survival rate corrected for intercurrent deaths was 57% (42–72%) among patients with pT1N1M0 ($n = 43$) or pT1N2M0 ($n = 3$) disease and 59% (43–75%) among the 43 patients with pT1N1M0 disease (Figure 3). Histological grade, the number of mitoses, presence of tumour necrosis, primary tumour size, axillary nodal status (pN2 vs pN1) and histological type were also significantly associated with corrected survival in a univariate analysis (Table 2). When these six factors classified as in Table 2 were entered into a multivariate analysis, only histological grade (relative risk 1.7; 95% confidence interval, 1.4–2.1), primary tumour size (1.8, 1.5–2.2) and axillary nodal status (2.0, 1.4–3.0) had independent influence on corrected or overall survival (Table 3).

When the series was divided into three cohorts based on the decade when the diagnosis was made, it turned out that survival rates were better in the 1960s and 1970s than in the 1940s and 1950s ($P < 0.0001$). The 15-year corrected survival rate found among women with pN+ disease diagnosed in the 1970s was 34% (25–43%) compared with 13% (6–20%) in the 1940s and 1950s (Table 4). The result remained essentially similar, if patients with pN2 disease were excluded from the analysis ($P < 0.0001$).

© *Cancer Research Campaign 1998*

**Table 2** Prognostic factors in a univariate analysis

| Factor | n | Corrected survival | | | | P |
|---|---|---|---|---|---|---|
| | | 5-year (%) | 10-year (%) | 20-year (%) | 30-year (%) | |
| Histological grade of differentiation | | | | | | |
| Well | 52 | 86 | 65 | 46 | 46 | |
| Moderately | 143 | 52 | 37 | 26 | 22 | |
| Poorly | 144 | 26 | 19 | 15 | 13 | < 0.0001 |
| Mitotic count/HPF[a] | | | | | | |
| ≤ 2 | 107 | 74 | 54 | 35 | 32 | |
| 2–3 | 133 | 37 | 26 | 22 | 18 | |
| > 3 | 99 | 27 | 21 | 17 | 14 | < 0.0001 |
| Presence of necrosis | | | | | | |
| No | 213 | 57 | 41 | 29 | 26 | |
| Yes | 126 | 28 | 21 | 16 | 14 | < 0.0001 |
| Tumour size | | | | | | |
| ≤ 2 cm | 46 | 73 | 63 | 57 | 57 | |
| 2–5 cm | 169 | 50 | 35 | 24 | 22 | |
| > 5 cm or pT4 | 118 | 28 | 17 | 12 | 10 | < 0.0001 |
| Axillary nodal status | | | | | | |
| pN1 | 304 | 49 | 36 | 27 | 24 | |
| pN2 | 35 | 17 | 7 | 0 | 0 | < 0.0001 |
| Histological type | | | | | | |
| Ductal | 291 | 42 | 31 | 22 | 19 | |
| Lobular | 35 | 58 | 33 | 28 | 21 | |
| Special | 13 | 100 | 89 | 67 | 67 | 0.006 |
| Age at diagnosis | | | | | | |
| ≤ 56 (median) | 171 | 39 | 26 | 23 | 20 | |
| > 56 | 168 | 53 | 42 | 24 | – | 0.09 |

[a]HPF, high-power field.

**Table 3** Multivariate survival analyses

| Factor | P | β | Standard error of β | Relative risk (95% confidence interval) |
|---|---|---|---|---|
| *Overall survival* | | | | |
| Histological grade (G3 vs G2 vs G1) | < 0.001 | 0.40 | 0.08 | 1.5 (1.3–1.8) |
| Primary tumour size (pT3–4 vs pT2 vs pT1) | < 0.001 | 0.39 | 0.09 | 1.5 (1.2–1.8) |
| Axillary nodal status (pN2 vs pN1) | < 0.001 | 0.76 | 0.19 | 2.1 (1.5–3.1) |
| Histological type | 0.21 | | | |
| Tumour necrosis | 0.50 | | | |
| Mitotic count | 0.79 | | | |
| *Corrected survival* | | | | |
| Histological grade (G3 vs G2 vs G1) | < 0.001 | 0.55 | 0.10 | 1.7 (1.4–2.1) |
| Primary tumour size (pT3–4 vs pT2 vs pT1) | < 0.001 | 0.58 | 0.10 | 1.8 (1.5–2.2) |
| Axillary nodal status (pN2 vs pN1) | 0.001 | 0.70 | 0.20 | 2.0 (1.4–3.0) |
| Histological type | 0.12 | | | |
| Tumour necrosis | 0.54 | | | |
| Mitotic count | 0.55 | | | |

**Table 4** Survival by the decade of diagnosis

| Time period | n | Survival corrected for intercurrent deaths | | | | |
|---|---|---|---|---|---|---|
| | | 5-year (%) | 10-year (%) | 15-year (%) | 20-year (%) | 30-year (%) |
| 1945–59 | 102 | 28 | 16 | 13 | 13 | 12 |
| 1960–69 | 96 | 52 | 36 | 30 | 26 | 24 |
| 1970–79 | 141 | 55 | 45 | 34 | 30 | – |

### DISCUSSION

In the present series a considerable proportion of breast cancer patients with histologically confirmed axillary nodal metastases at diagnosis were long-term survivors even if no systemic treatment was given. The series includes the great majority of patients diagnosed in a well-defined urban area, which precludes a major selection bias. Only a few deaths from ipsilateral breast cancer take place after the 15th year of follow-up in node-positive or node-negative disease (Hibberd et al, 1983; Nab et al, 1994; Joensuu and Toikkanen, 1995), and, therefore, it is unlikely that a longer follow-up would have changed the result of the study markedly.

These data suggest that involvement of the axillary lymph nodes by cancer is not a conclusive sign of disseminated disease. Hypothetically, the patients who survive for decades after the diagnosis of breast cancer metastatic to the axillary lymph nodes might have dormant cancer cells and die from intercurrent diseases before they succumb to breast cancer. However, we observed no deaths from ipsilateral breast cancer after the 24th year of follow-up, and the relative survival was the same as that found in the general population after the 15th year of follow-up. Therefore, locoregional therapy is likely to be curative in a subset of patients with node-positive breast cancer.

It is worth noting that patients with a small primary tumour (pT1N1M0) had as favourable as 59% 15-year survival rate corrected for intercurrent deaths. This figure is in line with that found by Rosen et al (1989), who estimated that 52% of patients with T1N1M0 disease might not have a recurrence within a nearly 20-year follow-up period. The long-term survival rate of 24% found in the present series treated with locoregional therapy in pN1-disease may be a conservative estimate regarding many women with pN1 breast cancer diagnosed at present, because more cancers with a small primary tumour and minimal axillary nodal involvement are now being diagnosed.

Although several conventional prognostic factors that correlated strongly with survival could be identified (Tables 2 and 3), none of the factors could identify a subset of patients with particularly favourable survival. Hence, adjuvant systemic therapy should probably be recommended to all patients with pN+ disease, because there is currently no reliable way to identify patients who might be cured without adjuvant therapy. However, the newer prognostic factors should be tested for this purpose.

In a randomized Danish study consisting of more than 1700 premenopausal patients with stage II or III breast cancer radiotherapy to the chest wall and the regional lymph nodes was found not only to reduce the frequency of locoregional relapses from 32% to 9%, but to increase significantly both disease-free and overall survival compared with patients who were treated with mastectomy and adjuvant chemotherapy alone (Overgaard et al, 1997). Similar results were also obtained in a randomized Canadian study (Ragaz et al, 1997). Both of these studies suggest that recurrent locoregional cancer may give rise to distant metas-

© Cancer Research Campaign 1998

tases in addition to the primary cancer, and that irradiation of the subclinical locoregional lymph node and chest wall metastases improves survival. These data also support the hypothesis that node-positive breast cancer has not always given rise to distant metastases, and that careful management of the regional lymph node regions is beneficial. Coupled with the present data, data from these randomized trials suggest that adequate locoregional therapy can cure many women with node-positive breast cancer.

We found better survival among the patients diagnosed in the 1960s and 1970s compared with those diagnosed earlier. The reasons for this remain speculative, but improved survival with time might in part be explained by reduction in breast cancer size and less extensive axillary nodal involvement in the 1970s compared with the 1940s and 1950s. However, improvements in surgical and radiotherapy techniques may also play a role. Modern locoregional radiotherapy improves survival in node-positive breast cancer, and orthovoltage and cobalt therapy used in the city in the 1940s to 1960s may have been inferior to radiotherapy given with linear accelerators in the 1970s. The rate of axillary nodal dissection did not increase consistently during the study period in the city (83%, 77% and 90% of patients had axillary nodal dissection in 1945–59, 1960–69 and 1970–79 respectively). Breast cancer, per se, might also have become less aggressive with time, but we consider this unlikely. When a few biological and histological prognostic factors were compared between breast cancers diagnosed in the city in the 1980s and those diagnosed in the 1940s to 1960s adjusting for the generally smaller size of the cancers diagnosed in the 1980s, little change in the malignancy grade with time could be found (Joensuu and Toikkanen, 1991).

In conclusion, the present results suggest that a subgroup of women with axillary nodal metastases from ipsilateral breast cancer are permanently cured by locoregional therapy alone. Therefore, axillary nodal involvement is not a conclusive sign of disseminated disease. Furthermore, the time trends in the study cohort suggest that the long-term survival rate of 24% among patients with pN1 disease and treated with locoregional therapy is a conservative estimate. The present results combined with those of recently published randomized trials suggest that careful locoregional treatment is mandatory in the care of node-positive breast cancer in addition to systemic therapy.

### ACKNOWLEDGEMENTS

The authors thank automatic data processing analyst Bengt Söderman from the Finnish Cancer Registry for calculation of the expected survival curves. Supported by the Cancer Society of Finland and Academy of Finland.

### REFERENCES

Adair F, Berg J, Joubert L and Robbins GF (1974) Long-term followup of breast cancer patients: the 30-year report. *Cancer* **33**: 1145–1150

Cancer Research Campaign Working Party (1980) Cancer Research Campaign (King's/Cambridge) trial for early breast cancer: a detailed update and the tenth year. *Lancet* **2**: 55–60

Fentiman I, Cuzick J, Millis RR and Hayward JL (1984) Which patients are cured of breast cancer? *Br Med J* **289**: 1108–1111

Fisher B, Redmond C, Fisher ER, Bauer M, Wolmark N, Wickerham L, Deutsch M, Montague E, Margolese R and Foster R (1985) Ten-year results of a randomised clinical trial comparing radical mastectomy and total mastectomy with and without radiation. *N Engl J Med* **312**: 674–681

Giuliano AE, Dale PS, Turner RR, Morton DL, Evans SW and Krasne DL (1995) Improved axillary staging of breast cancer with sentinel lymphadenectomy. *Ann Surg* **222**: 394–401

Harbeck N, Untch M, Pache L and Eiermann W (1994) Tumour cell detection in the bone marrow of breast cancer patients at primary therapy: results of a 3-year median follow-up. *Br J Cancer* **69**: 566–571

Hermanek P and Sobin LH (1992) *TNM Classification of Malignant Tumours*, (edn 4, 2nd revision). Springer-Verlag, Berlin: International Union Against Cancer

Hibberd AD, Horwood LJ and Wells JE (1983) Long term prognosis of women with breast cancer in New Zealand: study of survival to 30 years. *Br Med J* **286**: 1777–1779

Joensuu H and Toikkanen S (1991) Comparison of breast carcinomas diagnosed in the 1980s with those diagnosed in the 1940s to 1960s. *Br Med J* **303**: 155–158

Joensuu H and Toikkanen S (1995) Cured of breast cancer? *J Clin Oncol* **13**: 62–69

Lee CG, McCormick B, Mazumdar M, Vetto J and Borgen PI (1992) Infiltrating breast carcinoma in patients age 30 years and younger: long term outcome for life, relapse, and second primary tumours. *Int J Radiat Oncol Biol Phys* **23**: 969–975

Nab HW, Hop WCJ, Crommelin MA, Kluck HM, van der Heijden LH and Coebergh J-WW (1994) Changes in long term prognosis for breast cancer in a Dutch cancer registry. *Br Med J* **309**: 83–86

Overgaard M, Hansen PS, Overgaard J, Rose C, Anderson M, Bach F, Kjaer M, Gadeberg CC, Mouridsen HT, Jensen M-B and Zedeler K for the Danish Breast Cancer Cooperative Group 82b Trial (1997) Postoperative radiotherapy in high-risk premenopausal women with breast cancer who receive adjuvant chemotherapy. *N Engl J Med* **337**: 949–955

Ragaz J, Jackson SM, Plenderleith IH, Spinelli JJ, Basco VE, Wilson KS, Knowling MA, Coppin ML, Paradis M, Coldman AJ and Olivotto IA (1997) Adjuvant radiotherapy and chemotherapy in node-positive premenopausal women with breast cancer. *N Engl J Med* **337**: 956–962

Rosen PP, Groshen S, Saigo PE, Kinne DW and Hellman S (1989) A long-term follow-up study of survival in stage I (T1N0M0) and stage II (T1N1M0) breast carcinoma. *J Clin Oncol* **7**: 355–366

Rutqvist LE and Wallgren A (1985) Long-term survival of 458 young breast cancer patients. *Cancer* **55**: 658–665

Sacks NPM and Baum M (1993) Primary management of carcinoma of the breast. *Lancet* **324**: 1402–1408

World Health Organization (1981) *Histological Typing of Breast Tumours*, International Histological Classification of Tumours no. 2 (edn 2). Geneva: World Health Organization

© Cancer Research Campaign 1998